# Exploring the efficacy of plant-based nutraceuticals in managing diabetic neuropathy

**DOI:** 10.1007/s10787-025-01793-z

**Published:** 2025-05-28

**Authors:** Samea Khan, Maria Markoulli, Amy T. Tsoi, Mark Willcox

**Affiliations:** 1 School of Optometry and Vision Science, University of New South Wales, Sydney, NSW 2052 Australia; 2 School of Clinical Medicine, University of New South Wales, Sydney, NSW Australia

**Keywords:** Diabetic neuropathy, Plant extracts, Nutraceuticals, Animal models

## Abstract

Diabetes-induced peripheral neuropathy is one of the most common complications in patients with diabetes. Plant-derived natural products have been tested in animal models as agents to treat this complex condition. Therefore, this study aimed to examine the therapeutic potential of plant-based nutraceuticals used for managing diabetic neuropathy in rat models using a systematic literature review approach. The literature review was performed in November 2023 on two databases, PubMed and Scopus. Nerve conduction velocity and pain-related behaviors were considered primary outcome measures. For meta-analysis, a random effect model was selected with a mean difference at 95% confidence intervals to estimate the effect of plant-based nutraceuticals on the primary outcome measures. Eighteen studies were included in the meta-analysis, with results indicating that plant extracts or isolated compounds significantly increased nerve conduction velocity and thermal and mechanical paw withdrawal latencies. Different mechanisms of action to reduce neuropathy symptoms have been proposed in these studies including a reduction in inflammatory cytokines and oxidative stress markers. This study suggests that plant-based therapies are effective in the management of diabetes-induced peripheral neuropathy. However, more studies are required with a standard experimental design and reporting.

**Supplementary Information:**

The online version contains supplementary material available at 10.1007/s10787-025-01793-z.

## Introduction

Diabetic peripheral neuropathy, one of the most widespread microvascular complications of both type 1 and type 2 diabetes, restricts a patient’s quality of life and places a substantial burden on the healthcare system (Hosseini et al. 2022). According to the International Diabetes Federation, approximately one-third of the global population is predicted to suffer from diabetes by 2050, and half of those individuals will likely experience neuropathy without effective control of their diabetes (Boyle et al. 2010). Diabetic neuropathy is caused by the impairment of the sensorimotor and autonomic nerves in the peripheral nervous system due to hyperglycemia (Callaghan et al. 2015). It affects various sensory functions, such as loss of sensation in the feet, weakness of limbs, numbness, hyperalgesia, and allodynia in the feet (Çakici et al. 2016). However, previous studies have also suggested the contribution of other risk factors, such as dyslipidemia, hypertension, and obesity along with hyperglycemia for diabetic neuropathy development (Smith and Singleton 2013).

Despite the widespread prevalence and destructive effects of diabetic neuropathy, there is still no effective treatment that could potentially reduce the development and progression of this condition (Hosseini et al. 2022). Currently, approaches for managing diabetic neuropathy rely on glycemic control, modifications in lifestyle such as dietary interventions and exercise, and drug-based pain relief (Ang et al. 2018). Pathogenesis-based therapies include the use of glucagon-like peptide-1 (GLP-1) agonists, and sodium-glucose cotransporter (SGLT-2) inhibitors (Syed et al. 2023).

Due to the side effects associated with many pharmacological treatments, the use of plants and their extracts has emerged as an alternative approach for treating diabetes-linked complications (Oh 2016). The efficacy of traditional plants and plant parts has been investigated by various researchers for treating diabetes and its associated complications (Arora et al. 2021). This review aims to systematically evaluate the efficacy of plant-based nutraceuticals in alleviating diabetes-induced peripheral neuropathy in rat models.

## Methods

### Protocol registration

This review was registered in PROSPERO, the international database of a systematic review on (17/01/2024), and obtained the registration number CRD42024480420. The PRISMA (The Preferred Reporting Items for Systematic Reviews and Meta-Analyses) guidelines (Page et al. 2021) were used to construct this systematic review.

### PICOS strategy

This review was conducted under the PICOS strategy (da Costa Santos et al. 2007) providing the following information: P (problem), the peripheral neuropathy caused by diabetes; I (intervention,), treatment with plant-based nutraceuticals; C (control), standard drugs or without treatment; O (outcomes), pain-related behavior, nerve conduction velocities, peripheral nerve structure changes in animals with diabetes-induced peripheral neuropathy; S (type of study), pre-clinical studies.

### Literature search strategy

Two electronic databases, PubMed and Scopus, were systematically searched from the earliest available date to November 2023 for studies conducted on animal models. The following medical subject headings (MESH), keywords, and Boolean search criteria were used: [Diabetes Mellitus], OR [Diabetic Neuropathies], AND [Plant Extracts] OR [nutraceuticals], OR [Dietary Supplements]. Results were limited to studies published in English and conducted in animal models. No limits were applied to the publication date. The detailed search strategy is provided in supplementary file S1.

### Selection of studies

The selected databases were searched, and all records were imported into a web-based software (Covidence) for screening; the software automatically removed any duplicates. Two reviewers, Samea Khan (SK) and Maria Markoulli (MM), independently screened the titles and abstracts of the extracted articles to assess their suitability for inclusion in full-length article screening. The full texts were retrieved and evaluated based on the inclusion and exclusion criteria by SK and Amy Tsoi (AT). Any disagreement between the two reviewers was resolved by a third reviewer Mark Willcox (MW).

## Study inclusion criteria

### Types of studies

Studies investigating the effect of plant extracts and their isolated compounds on diabetic peripheral neuropathy in rat models were included.

### Animals

Only rat models of peripheral neuropathy induced by diabetes were included, as a preliminary review found that the majority of papers used this animal. The neuropathy assessment criteria in rodents established by “Neurodiab” a Diabetic Neuropathy Study Group in 2014 were followed (Biessels et al. 2014). The parameters of the presence of pain-related behaviors, nerve conduction velocities, and alterations in peripheral nerve structure were considered. Only those studies that met at least two out of these three parameters were included for screening.

### Interventions

Studies involving plant extracts or compounds isolated from plant extracts as an intervention were included.

### Study outcomes

Studies in reporting effects on pain-related behavior, such as hyperalgesia/or allodynia thermal or mechanical, as well as changes in nerve conduction velocities and alteration in peripheral nerve structure associated with diabetic peripheral neuropathy, were included.

### Data extraction

A standardized table extracted the following information from the selected studies: rat species, age, gender, and weight; peripheral neuropathy induction method; and behavioral tests for assessing neuropathy. Data about the plant extracts—plant type, dose, and route of administration—were also obtained.

### Statistical analysis

Review Manager 5.4 was used for meta-analysis of research data. The outcomes of pain-related behaviors and nerve conduction velocities were input as continuous variables. Heterogeneity among studies was measured via Chi^2^ statistics.

## Results

**Fig. 1:**
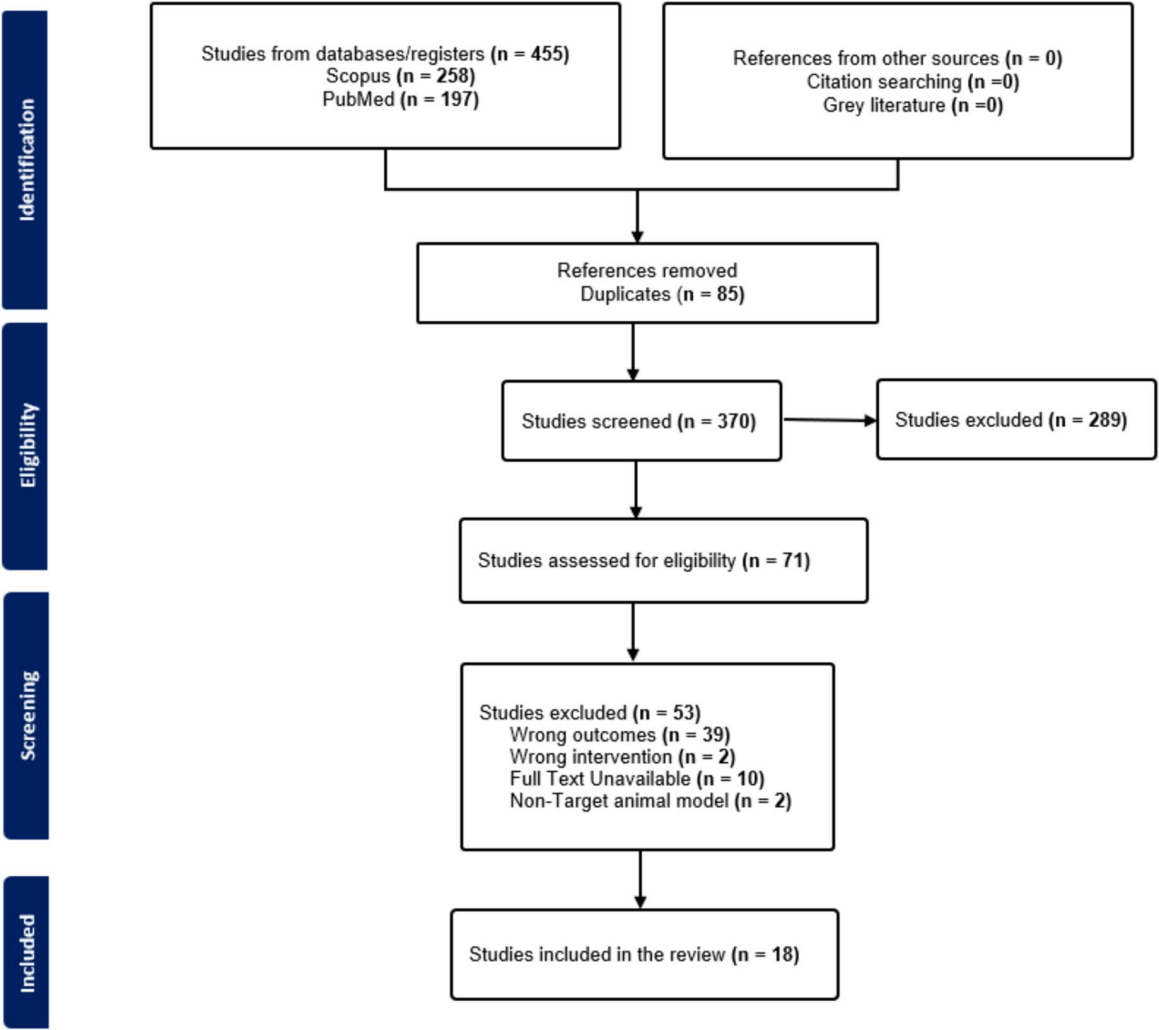
Prisma flowchart of the study identification and selection

Figure 1 presents a flowchart illustrating the identification and selection of studies. Initially, 455 records were retrieved by searching two databases (PubMed and Scopus) with keywords. A total of 85 duplicates were removed, and 289 studies were eliminated after the title and abstract screening. A total of 71 records were reviewed for full text, and 53 studies were excluded at this stage. Finally, 18 studies were included in the analysis.

## Study characteristics

Table 1 summarizes the characteristics of the included studies. Plant extracts used in studies were obtained by different methods, using different solvents. Nine studies (Han et al. 2014a; Kaur et al. 2017; Kishore et al. 2018; Lee et al. 2013; Wang et al. 2016; Wattanathorn et al. 2015; Yu et al. 2021; Zangiabadi et al. 2011a) reported the solvents used for the preparation of extract from the plants. Solvents used in these studies include ethanol, petroleum ether, chloroform, hydro alcohol, and hydroalcoholic solutions of varying concentrations and distilled water. Three studies (Cui et al. 2008a; Kandhare et al. 2012b; Zangiabadi et al. 2014b) mentioned the use of commercially available active compounds. While other studies did not mention the obtaining method. Details are shown in supplementary file S1.

**Table 1:** Characteristics of included studies

Article reference	Animal	Gender	Age (W) weight (g)	Standard drug	Intervention	Intervention Dose	Behavioral tests			Nerve conduction	Nerve structure	Other outcomes
							Thermal Pawa withdrawal	Tail flick latency	Mechanical paw withdrawal			
Lee 2013	SD rats	Male	6–8/160–180	NR	*Dioscorea japonica* + *Dioscorea nipponica Makino*	DA -9801 (extract mixture) 10, 50, 100 ng/kg/day	PWL (hot plate) high in treated rats		PWL (Von Frey) attenuated PWL in treated rats)		IENFD	
Kishore 2018	Wistar rats	Male	NR/280–300	Gabapentin, 30 mg/kg	*Eruca sativa* Mill	100, 200 and 400 mg/kg of EHA, 5 and 10 mg/kg of KP	PWL (hot plate) high in treated rats	TFL (hot water immersion) high in treated rats	PWL (m-von Frey) decrease in PWL was prevented in treated rats	Increase in MNCV after treatment		Decreased TBARS, Increased SOD and GSH
Kaur 2017	Wistar rats	Male	NR/260–280	Gabapentin, 30 mg/kg	*Dillenia indica* L. (*Dilleniaceae*)	100, 200 and 400 mg/ kg of DAE, g 5 and 10 mg/kg of CR	PWL (hot plate) high in treated rats	TFL (hot water immersion) high in treated rats	PWL (Von Frey) lower in DNPWL decrease inhibited in treated rats	Improvement in MNCV after treatment		Increased SOD, GSH, AGEs, Decreased TNF-α, TGF-β, and IL-1β
Laddha 2021	SD rats	Male	NR/180–200	Alpha lipoic acid	*Bauhinia variegata* Linn	AE and AlcE at the dose of 250, 500, and 1000 mg/kg	PWL (hot plate) high in treated rats	TFL (hot water immersion) high in treated rats	PWL (Von Frey) decrease inhibited in treated rats	Improvement in MNCV		Increase in SOD and CAT, decrease in MDA
Wang 2016	Wistar rats	Male	NR/180–220		*Toona sinensis*	ETS 0.10, 0.15, 0.2 g/kg/day		TFL (hot water immersion) lower in treated rats		Improvement in SNCV after treatment		Increase in SOD and GPX, decrease in MDA Decrease in NGF-β, TNF-α and IL-6
Ding 2014	SD rats	Male	NR/180–200	NR	Grapes (*Vitis*)	125, 250, and 500 mg/kg	extract treatment slightly prolonged PWL		PWL (Von Frey) lower in DNPWL decrease inhibited in treated rats	Improvement in SNCV after treatment	Little improvement in Sciatic-myelinated fiber damage	Reduced the concentration of free Ca2 + and elevated ATPase activities in treated rats
Wattanathorn 2015	Wistar rats	Male	NR/250–280	Gabapentin, 50 mg/kg, ascorbic acid at dose of mg/100 mg	*Zea mays* L. + *Zingiber officinale* Roscoe	ETS 0.10, 0.15, 0.2 g/kg/day	PWL (hot plate) increase in paw withdrawal thresh hold		PWL (Von Frey) attenuated PWL in treated rats	Increase in NCV	Increase in axonal density of myelinated fibers of sciatic nerve	Reduced MDA levels, Increased SOD, CAT, and GPX activity
CUI 2008	Wistar rats	Male	10/200–220		Grape (*Vitis*)	GSPE 250 mg/kg			PWL (m-von Frey) increased PWL in treated rats	Higher NCV Was Observed in treated rats	Improvement in sciatic axonal density, myelinated fibers and Schwann cells	Lower MDA and AGEs Increased SOD activity
Liu 2018	SD rats	Male	8/220 ± 20	Lipoic acid (LA)	*Lycium barbarum* polysaccharide (LBP)	LBP (500 mg/kg/d) and LA (100 mg/kg/d)	PWL (hot plate) increases in time in response to thermal stimuli		PWL (m-von Frey) ameliorated mechanical allodynia	Increased SNCV	Prevented sciatic nerve myelin and axonal injury	Inhibited the activation of the mTOR/p70S6K pathways
Liu 2010	SD rats	Male	NR/180–220		Tanshinone IIA	tanshinone IIA (20 mg/kg, 50 mg/kg and 100 mg/kg)	PWL (hot plate) Reversed the reduction of thermal nociception		PWT (m-von Frey) Increased mechanical nociception threshold	Increased MNCV		Increased SOD, CAT, and total antioxidant capacity Reduced MDA levels Increased nerve blood flow Increased Na + ,K + ATPase activity
Han 2014	SD rats	Male	NR/300–350		Aconite	FZE (1.75, 3.50, 7.00 g/kg)	PWL (hot plate) Delayed response time to thermal stimuli			Increased MNCV		Decreased BAX, and caspase3, 9 Increased BCL2
Hao 2017	SD rats	Male	8/250–300		cERPC Euonymus alatus, Radix trichosanthis, Panax notoginseng and Coptis chinensis	cERPC 15.4 g/kg 7.7 g/kg and 3.85 g/kg	PWL (hot plate) quicker response latency to thermal stimuli			Increased MNCV	Increased sciatic nerve axonal, and myelinated fiber area and diameter	Not Reported
Kandhare 2012	Wistar rats	Male	NR/150–200	insulin (10 IU/kg, s.c.)	Naringin	(20, 40 and 80 mg/kg)		TFL (hot water immersion test) significant increase in treated rats	PWT (m-von Frey) Increased mechanical nociception threshold	Increased MNCV	Not reported	Increased SOD and Na + ,K + ATPase Decreased LPO, NO, TNF-α
Zangiabadi 2014	Wistar rats	Male	6–8/250–300		*Melilotus officinalis*	5, 10, 20 mg/kg Angipars		TFL (Thermal Light) non-significant increase in TFL time		Improved MNCV	Increase in the axon, myelin sheath, and myelinated fiber diameter of sciatic nerve	Not Reported
Zangiabadi 2011	Wistar rats	Male	NR/250 ± 20	NR	Date Fruit (*Phoenix dactylifera*) Extract	4 ml/kg				Increased MNCV and SNCV		Not Reported
Liu 2011	Wistar rats	Male + Female	6/210 ± 10		Compounds of BAIMAI-SAN (ECGBM)	(LO) 0.1 mg/kg, (Mid) in which 0.3 mg/kg, (HI) in which 0.9 mg/kg ECGBM	PWL (Laser light) significantly increased response latency			Increased MNCV and SNCV		Not Reported
Yang 2015	SD rats	Male	NR/180–220	Alpha lipoic acid (20 mg/kg/day)	Tang Luo Ning Extract			TFL (Radiant heat) is significant decrease after treatment		Increased MNCV and SNCV		Decreased ROS, Bax/Bcl2 ratio, and cytochrome C Increased Nrf2and γGCS protein levels
Yu 2021	SD rats	Male	NR/280–300	EP epalrestat	Compound Xiong Shao Capsule		PWL (Radiant heat) significantly increased PWL		PWL (e-von Frey) Increased PWL	MNCV and SNCV significantly elevated	Prevented demyelination and Schwann cell structure	Reduced AGEs, elevated SOD, NOS Reduced caspase-3,BAX,and BCL2 expression^a^

^a^*SD* Sprague Dawley, *NR* No Record, *PWL* Paw Withdrawal Latency, *TFL* Tail Flick Latency, *MNCV* Motor Nerve Conduction Velocity, *SNCV* Sensory Nerve Conduction Velocity, *IENFD* Intra Epidermal Nerve Fiber Density, *MDA* Malondialdehyde, *SOD* Superoxide dismutase, *CAT* Catalase, *GSH* Glutathione, *GPX* Glutathione Peroxidase, *TBARS* Thiobarbituric acid reactive substances, *ROS* Reactive oxygen species, *AGEs* Advanced glycation End products, *TNF-α* Tumor Necrosis factor-α, *IL-1β* Interleukin beta, *IL-6* Interleukin- 6, *TGF-β*Transforming growth factor

Different approaches were used to induce diabetes in rats. Two species of rats were used in these studies, Sprague–Dawley (50%) and Wister rats (50%). All studies (18) used male rats except one (Liu et al. 2011), which included both male and female rats in their experiments. All studies described the weight of the rats, which ranged between 160 and 350 g. Only 6 studies (Cui et al. 2008b; Hao et al. 2017b; Lee et al. 2013; Liu et al. 2011, 2018; Zangiabadi et al. 2014a) mentioned the age of rats, which ranged between 6 and 12 weeks, while the remaining 12 studies (Ding et al. 2014; Han et al. 2014a; Kandhare et al. 2012a; Kaur et al. 2017; Kishore et al. 2018; Laddha et al. 2021; Liu et al. 2010; Wang et al. 2016; Wattanathorn et al. 2015; Yang et al. 2015; Zangiabadi et al. 2011b) mentioned the use of “adult rats”.

For the induction of diabetic neuropathy, various methods were used. Thirteen studies used a single intraperitoneal injection of streptozotocin (STZ) of different doses (45, 50, 55, 60, 65, and 70 mg/kg body weight) to induce diabetes. In three studies, a combination of diet and STZ was used for the induction of diabetes. However, the composition, duration of the diet, and dose of STZ were different in these studies. In one study, rats were fed a high-fat diet (HFD) for 6 weeks followed by 35 mg/kg STZ; in another study, rats were fed an HFD for 4 weeks followed by a high dose of STZ (60 mg/kg;); in the third study, rats were fed a high carbohydrate/high-fat (HCHF) diet for 5 weeks followed by 25 mg/kg STZ. Two studies administered, a high dose (65 mg/kg) of STZ after nicotinamide adenine dinucleotide (NAD) injection. The reported duration of diabetes ranged between 3 and 24 weeks.

Nerve conduction velocities were measured in all studies to assess neuropathy. Four studies reported both motor nerve conduction velocity (MNCV) and sensory nerve conduction velocity (SNCV). Seven studies used only MNCV, while the remaining five studies reported only SNCV.

Behavioral tests, including mechanical withdrawal threshold, thermal paw withdrawal latency, and tail-flick latency, were used. Twelve studies reported paw withdrawal latency threshold by the hot plate method. Nine of these studies reported hyperalgesia in diabetic rats, which means decreased paw withdrawal latency in diabetic rats as compared to normal rats, and an increase in latency duration after treatment with plant extracts. In hyperalgesia, noxious stimuli induce a painful response (Griggs et al. 2016). Three of the studies reported hypoalgesia with hot plate testing. Tail flick latency response is another method to measure thermal sensitivity (Tiwari et al. 2011). Tail flick latency was measured in six studies. Three of them used hot water tail immersion method, while the remaining three used radiant heat.

Mechanical sensitivity was assessed in a total of 11 studies all of which used von Frey filaments. Most studies reported the use of manual apparati; only one study used an electrical version. Responses were mostly expressed as paw withdrawal latency (PWL) in seconds. However, two studies presented their findings as the weight (in grams) of the von Frey element required to provoke a response.

Only one study reported measuring intraepidermal nerve fiber density (IENFD); other studies analyzed the morphometry or histology of peripheral nerves.

A few studies reported the use of any other substance as a positive control. The limited use of any other drug may be the result of the unavailability of suitable treatments for diabetic peripheral neuropathy (Santos et al. 2022).

Two studies (Zangiabadi et al. 2011b, 2014a) also performed open-field tests to investigate the effect of diabetes on rats' exploratory behaviors. The exploratory behaviors (Zangiabadi et al. 2014a) were composed of different aspects including frequency of grooming, rearing up, distance traveled in a horizontal direction, and duration of immobility in a defined area. No significant difference was observed in two aspects of the open-field test, velocity, and rearing among experimental groups. However, for grooming and duration of immobility, a significant effect of Angipars, an extract of *Melilotus officinalis*, was observed in the treated groups when compared with vehicle-treated diabetic group. In the other study (Zangiabadi et al. 2011a), rearing frequency and duration of immobility were significantly increased in the Date fruit (*Phoenix dactylifera* extract) treated groups. However, no difference was reported in grooming and total distance traveled by rats in both publications.

## Risk-of-bias assessment:

**Fig. 2:**
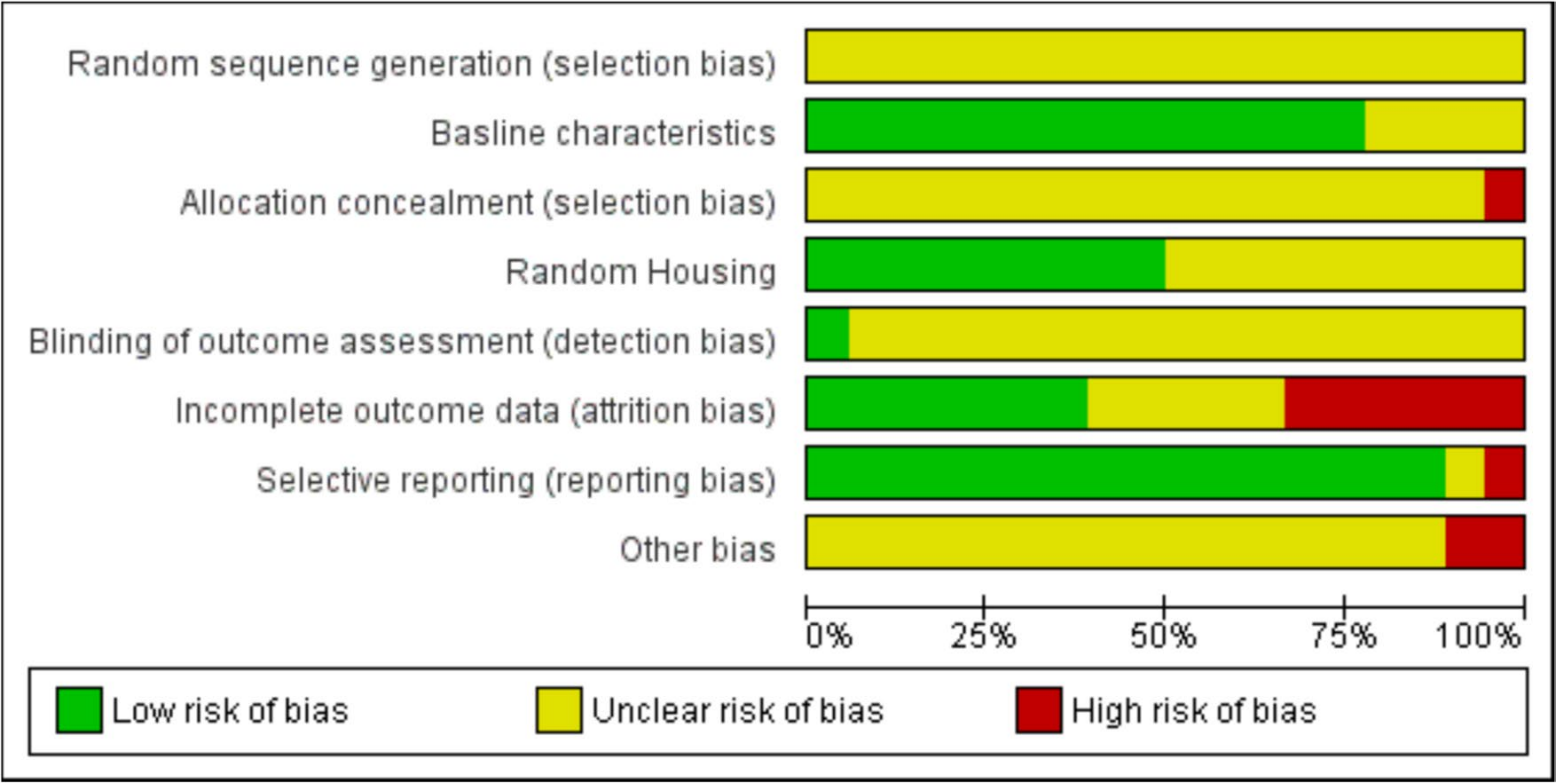
Graphical summary of the risk of bias for each study

The quality of the included studies was evaluated using the SYRCLE risk-of-bias tool designed for animal studies (Hooijmans et al. 2014) (Fig. 2). A total of eight domains were assessed; sequence generation, baseline characteristics, allocation concealment, random housing, blinding of caregivers, outcome assessor blinding, incomplete outcome data, selective outcome reporting, and other potential risks of bias such as funder influence on the results.

All domains were assessed as high, low, or unclear risk of bias. Responding “yes” indicated a high risk of bias, “no” indicated a low risk of bias, and “unclear” no adequate information to evaluate the risk of bias. The risk of selection bias was unclear in all studies. Most studies provided information about baseline characteristics, such as initial body weight, blood glucose levels, and other primary outcome measures, and reported no significant difference between control and experimental groups. However, a few studies (Ding et al. 2014; Laddha et al. 2021; Liu et al. 2011, 2010) did not provide adequate information. Allocation concealment was unclear in all studies except one (Hao et al. 2017a) which allocated animals into different groups based on their blood glucose levels and body weight. Half of the studies provided information about the random housing of animals. Blinding (masking) of outcome assessors was not clearly described in most studies, with only two studies (Lee et al. 2013; Liu et al. 2018) reporting that the outcome assessor was blinded to avoid the risk of bias. A high risk of bias was observed in incomplete outcome data (Ding et al. 2014; Hao et al. 2017a; Kandhare et al. 2012b; Liu et al. 2011, 2018; Yang et al. 2015; Zangiabadi et al. 2014b). A few studies (Liu et al. 2011, 2018) mentioned other risks of bias where the outcome measures were listed in the methods but not reported in the results. The risk of selective reporting was low in most of the studies; only one study (Liu et al. 2011) reported using selective data.

## Meta-analysis of the efficacy of efficacy of plant-based nutraceuticals in managing diabetic neuropathy

### Thermal withdrawal threshold

**Fig. 3:**
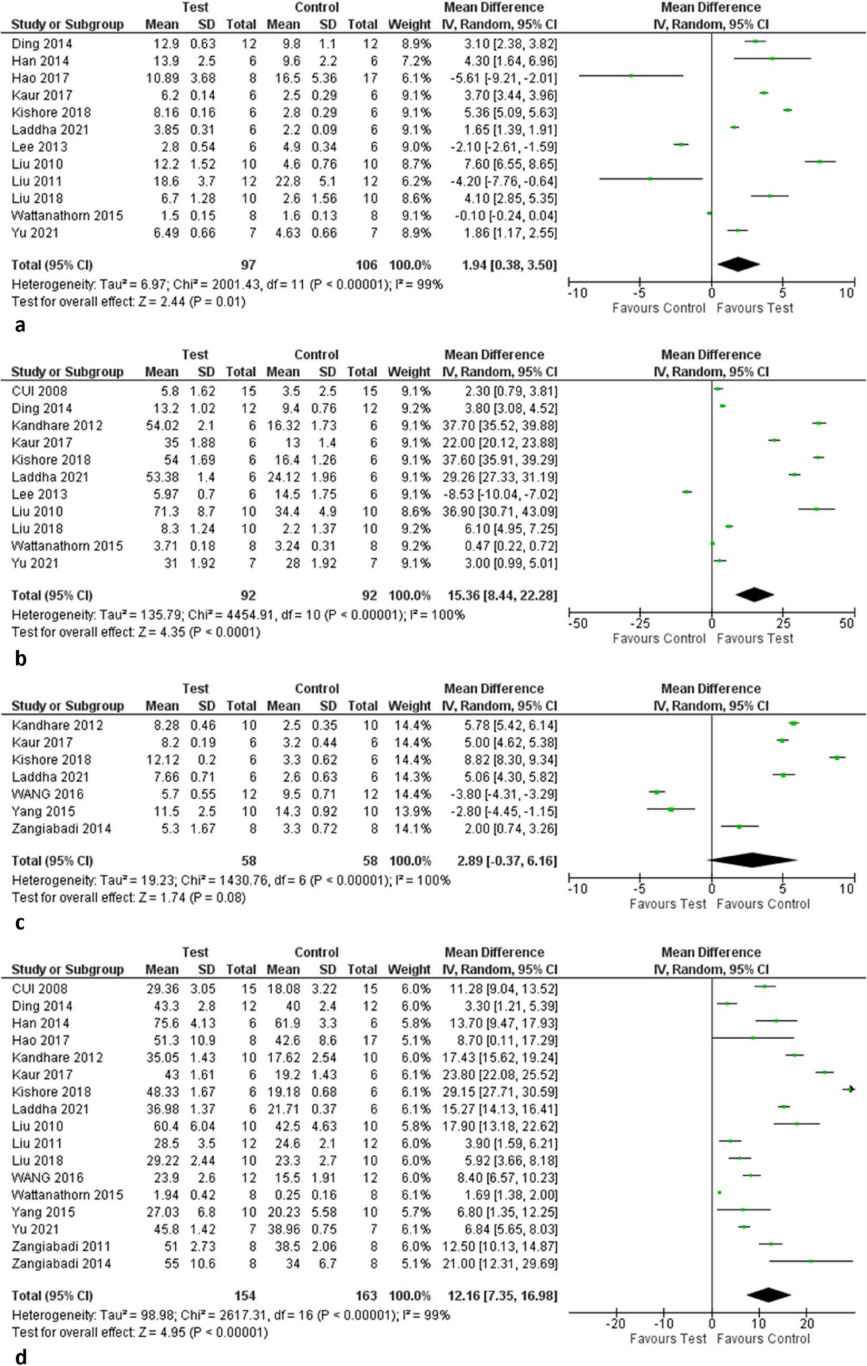
Metanalysis of Thermal Withdrawal Threshold (**a**), Mechanical Sensitivity Threshold (**b**), Tail Flick Latency (**c**), Nerve Conduction Velocity (**d**)

The results of a meta-analysis of thermal paw withdrawal latency are presented in Fig. 3a. Twelve studies were included in this analysis. Overall, plant extracts or isolated compounds elevated diabetes-induced changes in thermal pain perception in treated groups as compared to control groups (n = 203), with a mean difference of 1.94 [0.38, 3.50] at CI 95%, (P < 0.00001). With an I^2^ = 99%, there was considerable variation between the studies.

### Mechanical sensitivity threshold

The results of a meta-analysis of mechanical sensitivity measured by paw withdrawal latency are presented in Fig. 3b. The included studies (*n* = 11) found that plant extracts or isolated compounds significantly increased the response to the mechanical stimuli in treated groups as compared to control groups (*n* = 184), with a mean difference of 15.36 [8.44, 22.28] at CI 95%, (P < 0.00001). With an I^2^ = 100%, there was considerable variation between the studies.

### Tail flick latency

Seven studies with 116 rats were included in the meta-analysis of the tail-flick latency test (Fig. 3c). The results reported that the paw withdrawal latencies were significantly increased with plant extracts or isolated compounds in treated rats when compared with normal rats. The mean tail-flick latency was 2.89 (− 0.37 to 6.16) at 95% of CI (*P* < 0.00001), *I*^2^ = 100%.

### Nerve conduction velocity

Seventeen studies measured the nerve conduction velocities, see Fig. 3d. These studies found that plant extracts or isolated compounds significantly increased the nerve conduction velocities in treated groups as compared to control (*n* = 317), with a mean difference of 12.16 [7.35, 16.98] at CI 95% (*P* < 0.00001), *I*^2^ = 99%.

## Discussion

This systematic review and subsequent meta-analyses evaluated the effect of plant extracts or isolated compounds on diabetes-induced neuropathy in rat models. The results of the meta-analyses suggest that plant extracts and their isolated compounds increased the nerve conduction velocity which was reduced due to diabetes. There was an increase in thermal and mechanical paw withdrawal, as well as tail-flick latencies in diabetes-induced rat models treated with plant extracts. However, the meta-analyses found considerable variation between the studies, with I^2^ values ≥ 99%. Variations in the studies existed in the overall methodology of the experiments, such as the disease induction method, the treatment's start points, and the treatment duration.

The pathophysiology of diabetic neuropathy is complex and mechanisms behind it remain uncertain. Table 2 describes the possible mechanisms of plant extracts or their isolated active compounds in attenuating diabetic neuropathy in rats. These studies highlight the role of inflammatory cytokines, ion channels, oxidative stress, apoptotic pathways, nerve growth factors, and advanced glycation end products (AGEs). Upregulation of inflammatory cytokines is associated with reduced nervous system sensitivity, and targeting these proinflammatory pathways could be a potential therapeutic option to control disease progression (Liu et al. 2024). Four studies (Kandhare et al. 2012b; Kaur et al. 2017; Lee et al. 2013; Wang et al. 2016) reported decreased proinflammatory cytokine expressions, for instance, tumor necrosis factor-α (TNF-α), interleukin beta (IL-1β), interleukin- 6 (IL-6), and transforming growth factor (TGF-β) after using the plant extracts. Oxidative stress is caused by an imbalance between reactive oxygen species (ROS) production and antioxidant enzymes, which is a critical factor in the pathogenesis of diabetic peripheral neuropathy. Malondialdehyde (MDA), superoxide dismutase (SOD), catalase (CAT), glutathione (GSH), glutathione peroxidase (GPX), and thiobarbituric acid reactive substances (TBARS) are the key biomarkers associated with diabetic neuropathy (Zhang et al. 2020). Eight studies (Cui et al. 2008a; Kandhare et al. 2012b; Kishore et al. 2018; Laddha et al. 2021; Liu et al. 2010; Wang et al. 2016; Wattanathorn et al. 2015; Yu et al. 2021) have reported an increase in SOD, GSH, GPX, and CAT, and a decrease in MDA and TBARS after using plant extracts or their extracted compounds.

**Table 2:** Proposed mechanism of action in included studies

References	Intervention detail	Proposed mechanism
Lee et al. (2013)	Dioscorea japonica + Dioscorea nipponica Makino	Decrease in TNF-α and IL-6 increased NGF
Kishore et al. (2018)	Eruca sativa Mill	Decreased TBARS, Increased SOD and GSH
Kaur et al. (2017)	Dillenia indica L. (Dilleniaceae)	Decreased levels TNF-α, TGF-β, and IL-1β
Laddha et al. (2021)	Bauhinia variegata Linn	Increase in SOD and CAT, decrease in MDA
Wang et al. (2016)	Toona sinensis	Increase in SOD and GPX, decrease in MDA Decrease in NGF-β, TNF-α and IL-6
Ding et al. (2014)	Grapes	Reduced the concentration of free Ca2 + and ATPase activities in treated rats
Wattanathorn et al. (2015)	Zea mays L. + Zingiber officinale Roscoe	Reduced MDA levels, Increased SOD, CAT, and GPX activity
Cui et al. (2008b)	Grape	Decreased MDA, and AGEs, Increased SOD
Liu et al., (2018)	Lycium barbarum polysaccharide (LBP)	Inhibited the activation of the mTOR/p70S6K pathways
Liu et al. (2010)	Tanshinone IIA	Increased SOD, CAT, and total antioxidant capacity, Reduced MDA levels, Increased nerve blood flow, and Na + ,K + ATPase activity
Han et al. (2014b)	Aconite	Decreased BAX, and caspase3, 9, Increased BCL2
Hao et al. (2017b)	cERPC	Not Reported
Kandhare et al. (2012b)	Naringin	Increased SOD and Na + , K + ATPase, Decreased LPO, NO, TNF-α
Zangiabadi et al. (2014b)	Melilotus officinalis (Angipars)	Not Reported
Zangiabadi et al. (2011a)	Date Fruit Extract	Not Reported
Liu et al. (2011)	Compounds Groups of BAIMAI-SAN (ECGBM)	Not Reported
Yang et al., (2015)	Tang Luo Ning Extract	Decreased ROS, Bax/Bcl2 ratio, and cytochrome C Increased Nrf2and γGCS protein levels
Yu et al. (2021)	Compound XiongShao Capsule	Reduced AGEs, elevated SOD, NOS Reduced caspase-3 and BAX, increase in BCL2 expression^a^

^a^*MDA* Malondialdehyde, *SOD* Superoxide dismutase, *CAT* Catalase, *GSH* Glutathione, *GPX* Glutathione Peroxidase, *TBARS* Thiobarbituric acid reactive substances, *ROS* Reactive oxygen species, *AGEs* Advanced glycation End products, *TNF-α* Tumor Necrosis factor-α, *IL-1β* Interleukin beta, *IL-6* Interleukin- 6, *TGF-β* Transforming growth factor

Alterations in Na + , K + ATPase levels have been shown in the pathogenesis of diabetic peripheral neuropathy (Sachan et al. 2022). Two studies (Kandhare et al. 2012b; Liu et al. 2010) found increased Na + , and K + ATPase activity. Lower levels of serum AGEs were observed in three studies (Cui et al. 2008b; Kaur et al. 2017; Yu et al. 2021) after plant extract treatment. Oxidative stress can also trigger some apoptotic signaling pathways including BCL2, BAX, and caspase 3 and 9 (Neamatallah et al. 2018). Higher levels of apoptosis-related genes have been reported in nerve tissues of diabetic animal models (Zhu et al. 2018). Experiments in two studies (Han et al. 2014b; Yu et al. 2021) confirmed the increased BCL2 expression and reduced BAX and caspase 3 and 9. One study (Liu et al. 2018) highlighted the suppression of mTOR/ p70S6K pathways after the use of *Lycium barbarum* polysaccharide to promote autophagy. In 4 studies (Hao et al. 2017b; Liu et al. 2011; Zangiabadi et al. 2011a, 2014b), no mechanism or signaling pathway was reported.

There is no standard method documented for establishing diabetic peripheral neuropathy in rats. Various models are used to study the pathogenesis of the disease and evaluate suitable treatment options (Hossain et al. 2022). In the selected records for this systematic review, diabetes in rat models, either type I or type II, was induced by intraperitoneal injection of STZ. Diabetes-induced neuropathy was validated through various behavioral tests and nerve conduction velocities. In included studies, a type I diabetes model was used to illustrate diabetic neuropathy. Type I diabetes was induced by a single intraperitoneal injection of STZ at various concentrations ranging from 30 to 70 mg/kg of body weight. STZ has been widely used to induce diabetes in various animal models due to its ability to destroy pancreatic beta cells (Ghasemi and Jeddi 2023). However, the dose and duration of STZ are important to consider when modeling the specific type of diabetes. Single high or multiple low doses of STZ are believed to induce type I diabetes in rodents by beta cell destruction (Mostafavinia et al. 2016).

Type II diabetes in rats can be induced through diet, chemicals, genetic modifications, or a combination of these methods. Previous research has indicated that Type II diabetic rat models are more effective in studying neuropathy (Yorek 2022). STZ-induced diabetic models have been questioned due to a lack of morphological alterations in peripheral nerves (Wattiez et al. 2012). Many studies have utilized a combination of a high-fat diet (HFD) and STZ to induce obesity and insulin resistance, which mimic the conditions of Type II diabetes in humans (Gheibi et al. 2017). These studies support the use of the type II diabetic model in understanding the development of neuropathy and finding potential treatments (Coppey et al. 2020). Further research should focus on designing the DPN to achieve more reliable outcomes of treatments.

The “Neurodiab” guidelines (Biessels et al. 2014) established by a neuropathy study group in 2014 recommended the criteria for the assessment of neuropathy in rodent models. According to the guidelines, two out of three recommended parameters: nerve conduction, behavioral changes, and alterations in peripheral nerve structure should be incorporated into experimental designs to assess the development of neuropathy. Quantification of intraepidermal nerve fiber density (IENFD) along with behavioral assessment is considered the most important parameter for the assessment of DPN and the effectiveness of treatments (Rusli et al. 2024). In recent advancements in research, corneal confocal microscopy has emerged as a potential tool to visualize and quantify the small nerve fiber damage in the cornea in type II diabetes (Jin et al. 2021).

## Limitations

There are several limitations associated with this systematic review. Two electronic databases were systematically searched for literature, and there may be a possibility of missing related articles in the other databases. Most studies did not mention random sequence generation, allocation concealment, blinding of accessors, and other biases, which may lead to possible performance, selection, and detection bias. Studies with standardized experimental design and neuropathy assessment methods are required to reduce bias and heterogeneity in the studies conducted on rat models of diabetic neuropathy.

## Conclusion

Based on the meta-analysis results, we have found that plant-based nutraceuticals could increase nerve conduction velocity and improve diabetes-induced pain-related behaviors in rat models. However, further pre-clinical studies with improved experimental design and reporting are still required.

## Supplementary Information

Below is the link to the electronic supplementary material.

Supplementary file1 (DOCX 18 kb)

## Data Availability

Enquiries about data availability should be directed to the authors.
