# Supplementary material for: Exploring the efficacy of plant-based nutraceuticals in managing diabetic neuropathy
Source: Inflammopharmacology. 2025 May 28;33(6):2935–49. doi: 10.1007/s10787-025-01793-z (PMC12213865; doi:10.1007/s10787-025-01793-z)
Supplement: Supplementary file 1 — Supplementary file1 (DOCX 18 kb) [file 10787_2025_1793_MOESM1_ESM.docx]

# **Supplementary Material**

**Title:**

**Exploring the Efficacy of Plant-Based Nutraceuticals in Managing Diabetic Neuropathy**

Samea Khan,^1^ Maria Markoulli,^1^ Amy T Tsoi,^2^ Mark Willcox,^1*^

^1^ School of Optometry and Vision Science, University of New South Wales, Sydney, New South Wales, Australia

^2^ School of Clinical Medicine, University of New South Wales, Sydney, New South Wales, Australia

***Corresponding author:**

Professor Mark Willcox

School of Optometry and Vision Science, UNSW, Sydney, NSW 2052, Australia

Phone: +61290655394

Email: [m.willcox@unsw.edu.au](mailto:m.willcox@unsw.edu.au)

**Methods:**

**Search strategy.**

**Table S1: Search strategy used in Scopus, and PubMed databases.**

**Database: Scopus**

**No Query**

#1 (TITLE-ABS-KEY ( diabet* )

#2 TITLE-ABS-KEY ( neuropath* )

#3 TITLE-ABS-KEY ( "Plant extract*" OR nutraceutical* )

#4 TITLE-ABS-KEY ( "Dietary Supplement*" )

#1 AND #2 AND #3 OR #4

| Data Bases | Search Strategy | Results |
| --- | --- | --- |
| Scopus | ( TITLE-ABS-KEY ( diabet* ) AND TITLE-ABS-KEY ( neuropath* ) AND TITLE-ABS-KEY ( "Plant extract*" ) OR TITLE-ABS-KEY ( nutraceutical* ) OR TITLE-ABS-KEY ( "Dietary Supplement*" ) ) AND ( LIMIT-TO ( DOCTYPE , "ar" ) ) AND ( LIMIT-TO ( EXACTKEYWORD , "Nonhuman" ) OR LIMIT-TO ( EXACTKEYWORD , "Animal Model" ) ) AND ( LIMIT-TO ( LANGUAGE , "English" ) ) AND ( LIMIT-TO ( PUBSTAGE , "final" ) ) | **258** |

Search date: **28/09/2023**

**Database: PubMed**

**No Query**

#1 "Diabetes Mellitus"[Mesh]

#2 "Diabetic Neuropathies"[MeSH Terms]

#3 "Plant Extracts"[MeSH Terms]

#4 "Dietary Supplements"[MeSH Terms:noexp]

#5 "diabet*"[Title/Abstract]

#6 "neuropath*"[Title/Abstract]

#7 "plant extract*"[Title/Abstract]

#8 "nutraceutical*"[Title/Abstract]

#9 #1 OR #5 "Diabetes Mellitus"[MeSH Terms] OR "diabet*"[Title/Abstract]

#10 #2 OR #6 "Diabetic Neuropathies"[MeSH Terms] OR "neuropath*"[Title/Abstract]

#11 #9 AND #10 ("Diabetes Mellitus"[MeSH Terms] OR "diabet*"[Title/Abstract]) AND ("Diabetic

Neuropathies"[MeSH Terms] OR "neuropath*"[Title/Abstract])

#12 #3 OR #7 OR #8 OR #4 "Plant Extracts"[MeSH Terms] OR "plant extract*"[Title/Abstract] OR

"nutraceutical*"[Title/Abstract] OR "Dietary Supplements"[MeSH Terms:noexp]

#13 #11 AND #12 (("Diabetes Mellitus"[MeSH Terms] OR "diabet*"[Title/Abstract]) AND ("Diabetic Neuropathies"[MeSH Terms] OR "neuropath*"[Title/Abstract]) AND ("Plant Extracts"[MeSH Terms] OR "plant extract*"[Title/Abstract] OR "nutraceutical*"[Title/Abstract] OR "Dietary Supplements"[MeSH Terms:noexp])) AND ((fft[Filter]) AND (animal[Filter]) AND (english[Filter]))

| Data Bases | Search Strategy | Results |
| --- | --- | --- |
| PubMed | (("Diabetes Mellitus"[MeSH Terms] OR "diabet*"[Title/Abstract]) AND ("Diabetic Neuropathies"[MeSH Terms] OR "neuropath*"[Title/Abstract]) AND ("Plant Extracts"[MeSH Terms] OR "plant extract*"[Title/Abstract] OR "nutraceutical*"[Title/Abstract] OR "Dietary Supplements"[MeSH Terms:noexp])) AND ((fft[Filter]) AND (animal[Filter]) AND (english[Filter])) | 197 |
